# Validation of genes affecting rice mesocotyl length through candidate association analysis and identification of the superior haplotypes

**DOI:** 10.3389/fpls.2023.1194119

**Published:** 2023-05-30

**Authors:** Yamei Wang, Hongyan Liu, Yun Meng, Jindong Liu, Guoyou Ye

**Affiliations:** ^1^ Institute of Crop Sciences, National Wheat Improvement Center, Chinese Academy of Agricultural Sciences (CAAS), Beijing, China; ^2^ CAAS-IRRI Joint Laboratory for Genomics-Assisted Germplasm Enhancement, Agricultural Genomics Institute at Shenzhen, Chinese Academy of Agricultural Sciences, Shenzhen, China; ^3^ School of Agriculture, Sun Yat-sen University, Shenzhen, China; ^4^ Sanya Nanfan Research Institute of Hainan University, Hainan University, Sanya, China; ^5^ Strategic Innovation Platform, International Rice Research Institute, Manila, Philippines

**Keywords:** candidate gene association analysis, mesocotyl, haplotype, mr-MLM, Oryza sativa L

## Abstract

Mesocotyl is an essential organ of rice for pushing buds out of soil and plays a crucial role in seeding emergence and development in direct-seeding. Thus, identify the loci associated with mesocotyl length (ML) could accelerate breeding progresses for direct-seeding cultivation. Mesocotyl elongation was mainly regulated by plant hormones. Although several regions and candidate genes governing ML have been reported, the effects of them in diverse breeding populations were still indistinct. In this study, 281 genes related to plant hormones at the genomic regions associated with ML were selected and evaluated by single-locus mixed linear model (SL-MLM) and multi-locus random-SNP-effect mixed linear model (mr-MLM) in two breeding panels (Trop and Indx) originated from the 3K re-sequence project. Furthermore, superior haplotypes with longer mesocotyl were also identified for marker assisted selection (MAS) breeding. Totally, *LOC_Os02g17680* (explained 7.1-8.9% phenotypic variations), *LOC_Os04g56950* (8.0%), *LOC_Os07g24190* (9.3%) and *LOC_Os12g12720* (5.6-8.0%) were identified significantly associated with ML in Trop panel, whereas *LOC_Os02g17680* (6.5-7.4%), *LOC_Os04g56950* (5.5%), *LOC_Os06g24850* (4.8%) and *LOC_Os07g40240* (4.8-7.1%) were detected in Indx panel. Among these, *LOC_Os02g17680* and *LOC_Os04g56950* were identified in both panels. Haplotype analysis for the six significant genes indicated that haplotype distribution of the same gene varies at Trop and Indx panels. Totally, 8 (*LOC_Os02g17680-Hap1* and *Hap2*, *LOC_Os04g56950-Hap1*, *Hap2* and *Hap8*, *LOC_Os07g24190-Hap3*, *LOC_Os12g12720-Hap3* and *Hap6*) and six superior haplotypes (*LOC_Os02g17680-Hap2*, *Hap5* and *Hap7*, *LOC_Os04g56950-Hap4*, *LOC_Os06g24850-Hap2* and *LOC_Os07g40240-Hap3*) with higher ML were identified in Trop and Indx panels, respectively. In addition, significant additive effects for ML with more superior haplotypes were identified in both panels. Overall, the 6 significantly associated genes and their superior haplotypes could be used to enhancing ML through MAS breeding and further promote direct-seedling cultivation.

## Introduction

Rice (*Oryza sativa*) is one of the most important food crops in the world. Maintaining a higher and stable grain yield is crucial for food security especially in developing countries of Asia, such as China, Philippines, Vietnam and Malaysia. Traditional transplanting and direct-seeding are two major patterns for rice. Direct-seeding without transplanting process is labor-saving and water-efficient (Kumar and Ladha, 2011; Kato and Katsura, 2014; Liu et al., 2015; Ohno et al., 2018; Zhan et al., 2020). However, there are many disadvantages for direct-seeding, such as low seeding emergence rate, poor seeding establishment, weed infestation and high crop lodging rate (Mahender et al., 2015; Lee et al., 2017). Mesocotyl, an organ developed during rice seed germination in the dark and connects the coleoptile node and the basal part of seminal root, plays a key role in pushing buds out of deep water for successful seeding establishment (Zhan et al., 2020). Therefore, varieties with longer mesocotyl could be used to solve the problems induced by direct seeding cultivation (Lee et al., 2017; Zhan et al., 2020).

ML is a typical quantitative trait controlled by minor genes (Wu et al., 2015; Sun et al., 2018; Liu et al., 2020; Zhan et al., 2020; Jang et al., 2021; Zhang et al., 2022). Up to now, over 40 ML related Quantitative trait loci (QTLs) have been identified on 12 chromosomes and explain 5.7-27.8% of the phenotypic variations (Liu et al., 2020; Rohilla et al., 2020; Zhan et al., 2020). Recent advances in rice functional genomics facilitated the cloning and functional characterization of ML related genes, including *GY1* (Xiong et al., 2017), *OsGSK2* (Sun et al., 2018), *OsSMAX1* (Zheng et al., 2020) and *OsPAO5* (Lv et al., 2021). All the above cloned genes are involved in the plant hormone regulation. Also, previously reports showed that the mesocotyl elongation is regulated by various plant hormones, including Auxin (IAA), gibberellins (GA), ethylene (ETH), cytokinin (CTK) (Yuldashev et al., 2012), abscisic acid (ABA) (Watanabe and Takahashi, 1999; Watanabe et al., 2001; Wu et al., 2002), Jasmonic acid (JA), Strigolactones (SL) (Hu et al., 2014) and Brassinolide (BR). The mutual regulation of various plant hormones jointly regulates the elongation of rice mesocotyl (Xiong et al., 2017). Of these, IAA, GA, ETH, CTK and ABA can promote mesocotyl elongation; whereas JA and SL plays an inhibitory role. Lower concentration BR promotes mesocotyl elongation, whereas higher concentration inhibits. GA promotes cell elongation by changing the arrangement direction of cell microtubules and enhancing pectin methylation (Watanabe et al., 2001), whereas IAA mainly upregulates the activity of cell wall relaxant enzyme and promote cell growth (Zhan et al., 2020). ABA promotes the elongation of mesocotyl by inhibiting BR signaling pathway and then enhancing cell division near coleoptile node (Wu et al., 2002); whereas ETH promotes mesocotyl elongation by inhibiting JA synthesis (Xiong et al., 2017).

Association analysis is a powerful approach to understand clearly the genetic mechanism for complex traits (Flint-Garcia et al., 2003; Zhu et al., 2008; Wen et al., 2018). Single-locus mixed linear model (SL-MLM) is the most commonly used association analysis method, which was influenced seriously by polygenic background, including population structure and kinship (Zhu et al., 2008; Cui et al., 2018; Zhang et al., 2020a). Multi-locus association analysis (ML-AA), a method solves the SL-MLM induced shortcomings by estimating all the genetic effects across all the whole genome (Wen et al., 2018; Zhang et al., 2020b). ML-AA outperformed single locus-based methods in identify the minor effects loci of quantitative inheritance crop complex traits (Tamba and Zhang, 2018; Wen et al., 2018; Yang et al., 2020). Candidate gene association study (CAS) based on the target genes at the functional regions further increased the mapping resolution (Flint-Garcia et al., 2003; Zhu et al., 2008). Identifying minor genes of complex traits by CAS were conducted in *Arabidopsis*, rice, maize and common wheat (Zhao et al., 2015). Haplotype is the combination of alleles at different position on the same genomic regions for common inheritance (Liu et al., 2021), effective than SNP (Single nucleotide polymorphism) and InDel (Insertion-deletion) in crop marker-assisted selection (MAS) breeding (Li et al., 2017; Resende et al., 2017; Prodhomme et al., 2020). Superior haplotype identification has been proven to be an effective way to identify genes associated with complex traits and availability for crop breeding (Bevan et al., 2017; Abbai et al., 2019; Sinha et al., 2020). Previous approaches for genetic studies of ML were mainly focused on traditional linkage or association mapping, which hardly evaluate the existence and effects of natural variants and haplotypes. Thus, evaluating the genetic effects of the candidate gene for ML by CAS and identifying its correspondence superior haplotype in natural populations will accelerate the genetic improvement of ML.

Until now, substantial MAS breeding practices have been conducted to disease resistance, abiotic stress tolerance and yield related traits (Wang et al., 2020). However, MAS for ML is hindered due to the rare details of ML related genes and their haplotypes. To promote the progress of rice higher ML breeding, the effects of 281 selected genes related to plant hormones at reported ML genomic regions were evaluated in two breeding panels and the corresponding superior haplotypes were identified (**Tables 1**, **S1**).

**Table 1:** The reported genetic regions for mesocotyl length in rice.

Number	Chromosome	Start (Mb)	End (Mb)	Reference
1	1	0.3	2.3	Wu et al., 2015; Zhao et al., 2018
2	1	6.6	8.1	Wang et al., 2021
3	1	9.4	11.7	Jang et al., 2021
4	1	14.1	17.3	Lu et al., 2016; Liu et al., 2020; Jang et al., 2021; Wang et al., 2021
5	1	18.5	20.4	Wu et al., 2015; Wang et al., 2021
6	1	36.6	39	Xiong et al., 2017; Wang et al., 2021
7	1	40.4	40.5	Wu et al., 2015; Jang et al., 2021; Wang et al., 2021;
8	2	5.6	8.7	Zhao et al., 2018; Liu et al., 2020;
9	2	10	10.9	Jang et al., 2021
10	2	11.7	15.4	Liu et al., 2020
11	2	24	24.5	Jang et al., 2021
12	2	30.5	30.6	Jang et al., 2021
13	3	9	11	Jang et al., 2021
14	3	15.2	15.3	Liu et al., 2020
15	3	25.1	27.5	Wu et al., 2015; Wang et al., 2021
16	3	28.9	32.3	Zhao et al., 2018; Wang et al., 2021; Liu et al., 2021; Jang et al., 2021
17	3	34.1	34.1	Jang et al., 2021; Zhang et al., 2022
18	3	35.7	36.2	Wang et al., 2021
19	4	9.1	9.2	Wu et al., 2015; Jang et al., 2021
20	4	16	16.7	Jang et al., 2021
21	4	19.6	21.9	Wang et al., 2021
22	4	25.5	27.8	Wang et al., 2021; Zhang et al., 2022
23	4	32.4	34.8	Lu et al., 2016
24	5	3.2	3.8	Jang et al., 2021
25	5	5.8	6.3	Ouyang et al., 2005; Sun et al., 2018; Liu et al., 2020
26	5	9	12	Liu et al., 2020; Jang et al., 2021; Wang et al., 2021
27	6	2.6	5.1	Wang et al., 2021
28	6	7.3	9.7	Liu et al., 2020; Huang et al., 2010
29	6	15.3	16.6	Liu et al., 2020
30	6	23.3	24.9	Wu et al., 2015; Jang et al., 2021
31	6	30.3	31.4	Wu et al., 2015; Jang et al., 2021
32	7	3.8	8.7	Jang et al., 2021; Wang et al., 2021
33	7	10	13.7	Zhao et al., 2018; Liu et al., 2020; Wang et al., 2021
34	7	14.6	15.6	Zhao et al., 2018; Wang et al., 2021
35	7	16.1	18.5	Zhao et al., 2018; Wang et al., 2021
36	7	23.8	24.6	Zhao et al., 2018; Wang et al., 2021
37	8	2.8	5.2	Liu et al., 2020
38	8	9.1	10.6	Li et al., 2017; Jang et al., 2021
39	9	1.3	2.8	Lu et al., 2016; Liu et al., 2020
40	9	6.6	7.1	Wu et al., 2015; Zhao et al., 2018
41	9	9.1	10.3	Liu et al., 2020; Jang et al., 2021; Wang et al., 2021;
42	9	12	13.5	Zhao et al., 2018
43	11	0.9	1.2	Jang et al., 2021
44	11	6	6.1	Zhao et al., 2018
45	11	10.1	10.2	Jang et al., 2021; Zhang et al., 2022
46	11	23.5	26.8	Jang et al., 2021
47	12	0.6	0.8	Jang et al., 2021
48	12	4.4	4.5	Liu et al., 2020
49	12	6	7.8	Liu et al., 2020
50	12	13.3	15	Lee et al., 2012; Liu et al., 2020

## Materials and methods

### Plant materials

Two breeding populations (Trop and Indx) originated from the 3K re-sequence projects were employed in this study (Li et al., 2014; Alexandrov et al., 2015; Wang et al., 2018). The Trop panel including 331 *Japonica* (*Geng*) accessions mainly from Malaysia, United States, Philippines and Indonesia; whereas Indx panel including 470 *Indica* (*Xian*) accessions mainly from India, Philippines, China, Myanmar and Indonesia. The selected accessions have higher genetic polymorphism with various background.

### Genotyping, population structure and haplotype analysis

All the genotypes of Trop and Indx panels were obtained from the 3K-resequence projects (https://snp-seek.irri.org/_snp.zul) (https://www.rmbreeding.cn/) (Alexandrov et al., 2015; Wang et al., 2018). Sequence reads (nearly 12×) were aligned to the Nipponbare RefSeq (IRGSP-1.0) (http://rice.plantbiology.msu.edu/index.shtml). The variants for each accession were called by the GATK V3.2.2. Stringent filtering strategy was conducted (QUAL < 30.0, QD < 10.0, FS > 200.0, MQRankSum < -12.5 and ReadPosRankSum < -8.0). In the present study, markers with minor allele frequency (MAF) < 0.05 and missing rate > 0.05 were removed. SNPs and InDels were annotated by ANNOVAR (Wang et al., 2010). The SNPs and Indels located in the CDS region and the promoter (-1500 bp) of the 281 selected genes were extracted and used for further CAS and haplotype analysis. Haplotype analysis was conducted by considering the nonsynonymous variations at RFGB database (https://www.rmbreeding.cn/). The SNPs for haplotype analysis were filtered according to the following requirements: (1) only two alleles; (2) missing data < 0.1; (3) MAF ≥ 0.05; (4) exclude the correlated markers (*r* ^2^ = 1.0).

### Phenotyping of ML and seedling height

The ML were measured according to Wang et al. (2021). In short, 15 plump seeds for each accession were sown in a plastic tray with nutrient soil at 6 cm), then the plastic tray was placed in a pallet with nutrient soil at 3 cm. The whole devices were then kept in a dark incubator (30°C/65% RH) for about 10 days after all seeds germinated. Seedings were carefully excavated and washed with ddH_2_O for ML measurement by Image J (https://imagej.en.softonic.com/). The mean of two replications was calculated as the phenotype data for further CAS analysis. The seedling height for the accessions from Trop and Indx were originated from the RFGB database (Wang et al., 2018) (https://www.rmbreeding.cn/).

### Candidate gene study and superior haplotype identification

CAS was carried out using the Tassel V5.1 with a mixed linear model accounting for both PCA and kinship (Bradbury et al., 2007; Lipka et al., 2012). The Manhattan and QQ plots were drawn by CMplot (https://github.com/YinLiLin/CMplot) based on R v3.6.4. Mr-MLM V2.1, was used to perform the mr-MLM algorithm (Wang et al., 2020). The threshold for marker-trait association (MTA) was set as *P*>10^-4^ in SL-MLM and at a LOD value of 3.0 in mr-MLM. The significant genes were further used to identify superior haplotypes by Duncan analysis of ML means at Trop and Indx. Furthermore, only haplotypes existed at least five accessions in each panel were included for statistical analysis to ensure the accuracy of the results.

## Results

### Phenotype and genotype analysis

Continuous variation with transgressive segregation on both sides for ML and seedling height were observed across both Trop and Indx panels with approximately normal distributions (**Figure S1**). The ML for the Trop panel ranged from 0.20 to 4.40 cm with an average of 1.81 cm (**Table S2**), whereas the data ranged from 0 to 4.60 cm with an average of 1.40 cm for Indx panel (**Table S3**). The standard deviation and coefficient of variation of ML were 0.899 cm (coefficient of variation 0.50) and 0.893 cm (coefficient of variation 0.659) of Trop and Indx panel, respectively. The seedling height for the Trop panel ranged from 12.0 to 61.0 cm with an average of 34.7 cm (**Table S2**), whereas the data ranged from 16.0 to 74.0 cm with an average of 40.9 cm for Indx panel (**Table S3**). The standard deviation and coefficient of variation of seedling height were 10.4 cm (coefficient of variation 0.30) and 11.8 cm (coefficient of variation 0.29) of Trop and Indx panel, respectively.

**Figure 1:**
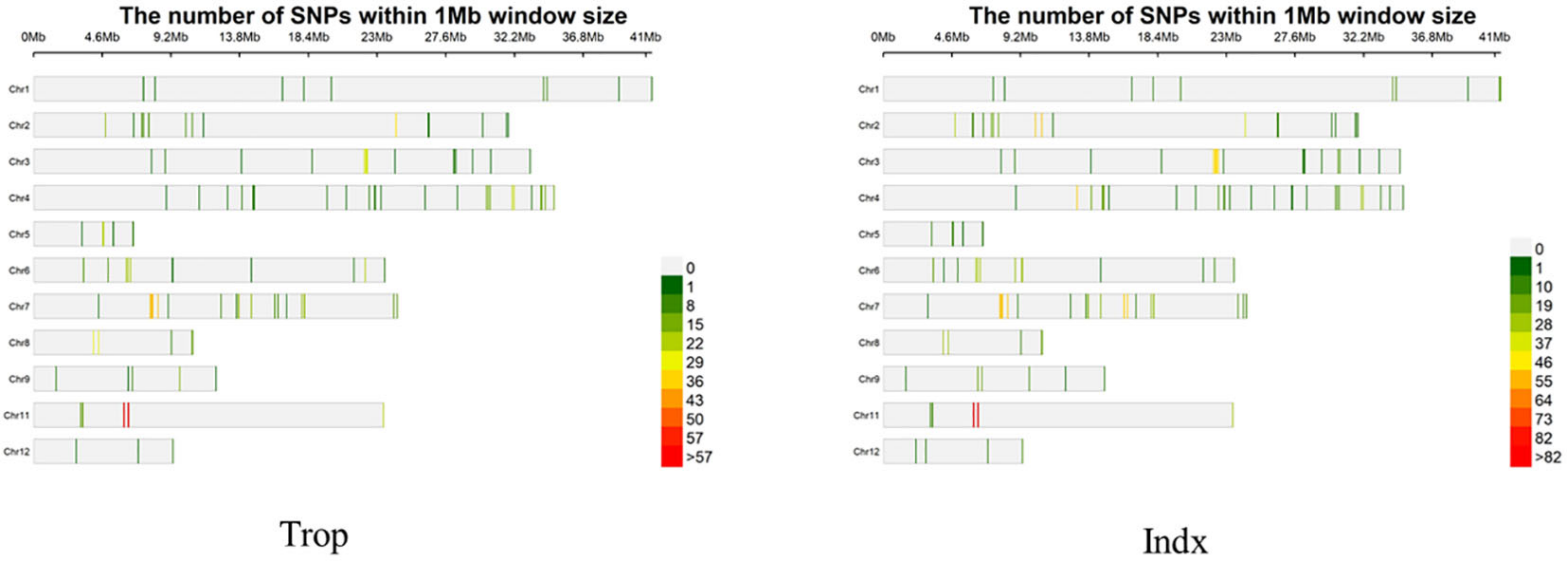
Marker distribution in Trop and Indx panels. Trop pane, Indx panel.

A total of 2277 SNPs and 1414 Indels were identified in the CDS and promoter regions of 281 selected genes in both panels. The SNPs and Indels for each gene ranged from 5 to 25 with the mean at 8.10 and 0 to 14 with the mean at 5.03 (**Tables S4**, **S5**; **Figure 1**). Trop and Indx subpopulation were classified in the 3K Rice Genomes Project and could be related to the geographic origins (Wang et al., 2018). Thus, population structure analysis was not conducted in this study and the MLM model were used for further CAS analysis. Principal component analysis indicated that the total variation explained by the top three PCs were 28.5%, 8.2% and 3.2% in Trop panel, whereas 24.5%, 9.2% and 7.3% in Indx panel.

### SL-AA and ML-AA analysis

**Figure 2:**
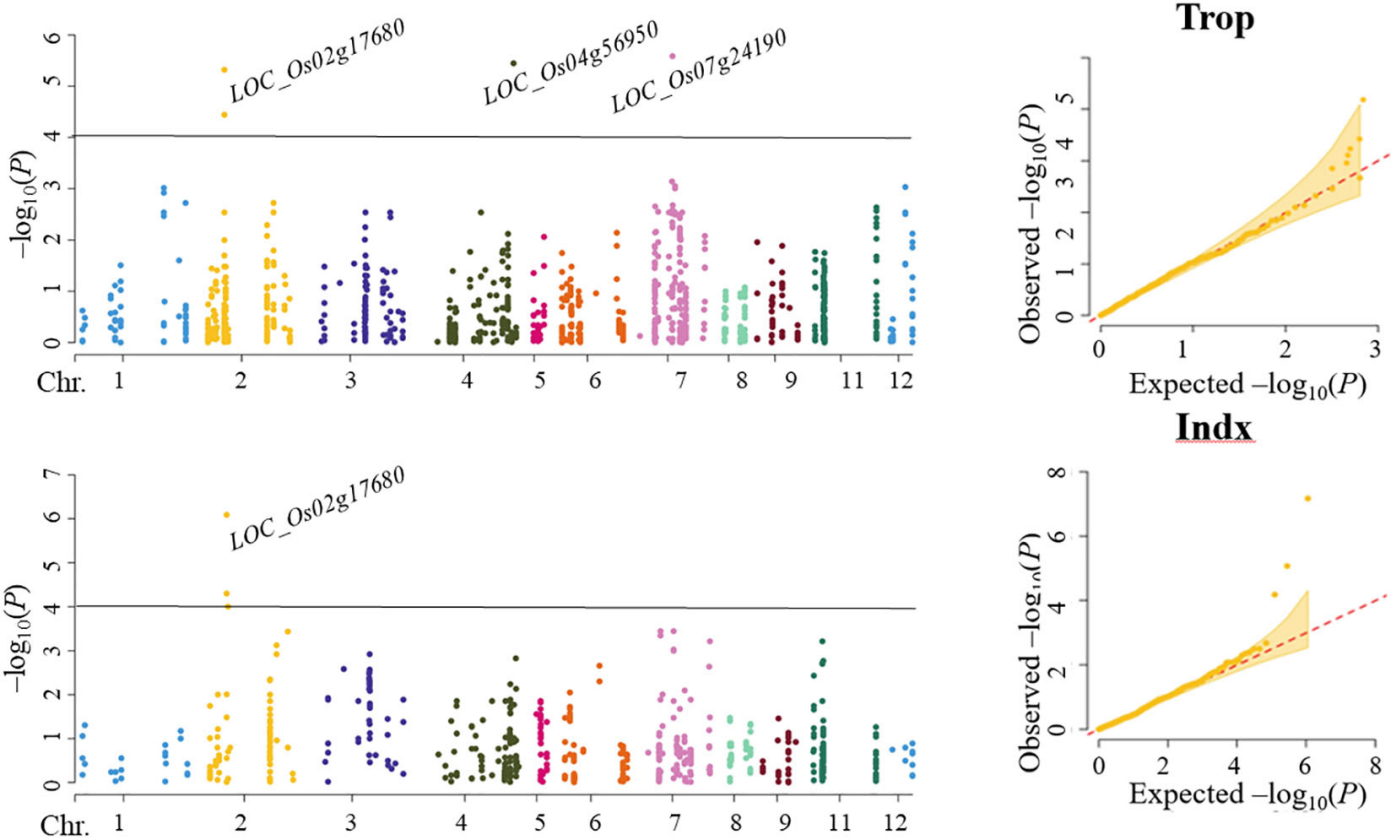
Association analysis for mesocotyl length content by SL-MLM in Trop and Indx panels.

In Trop, four SNPs corresponding to *LOC_Os02g17680* (including 2 SNPs), *LOC_Os04g56950* and *LOC_Os07g24190* were found to be significantly associated with ML by SL-MLM, and each explained the phenotypic variation of 5.6-7.6%, 8.0% and 8.1%, respectively (**Figure 2**; **Table 2**). As shown by SL-MLM, only *LOC_Os02g17680* was significantly associated with ML in the Indx and explained phenotypic variations of 5.4-8.2%, respectively (**Figure 2**; **Table 2**). For mr-MLM, eight significant SNPs (LOD ≥ 3.0) corresponding to four candidate genes (*LOC_Os02g17680*, *LOC_Os04g56950*, *LOC_Os07g24190* and *LOC_Os12g12720*) were simultaneously found to be significantly associated with the ML in Trop and explained phenotypic variation ranging from 5.6-9.3% (**Table 2**). Mr-MLM showed that six significant SNPs corresponding to four genes (*LOC_Os02g17680*, *LOC_Os04g56950*, *LOC_Os06g24850* and *LOC_Os07g40240*) were significantly associated with ML in the Indx panel and explained phenotypic variations of 6.5-7.4% (2 SNPs), 5.5%, 4.8% and 4.8-7.1% (2 SNPs), respectively (**Table 2**).

**Table 2:** List of detected mesocotyl length associated genes in Trop and Indx panels.

Population	Candidate gene	Chromosome	Start (bp)	End	Position (bp)	SL-MLM		Mr-MLM	
				(bp)		P-value	r^2^ (%)	LOD score	r^2^ (%)
Trop	*LOC_Os02g17680*	2	10181426	10189201	1E+07	4.80E-06	7.6	5.42	8.9
Trop	*LOC_Os02g17680*	2	10181426	10189201	1E+07	3.60E-05	5.6	4.23	7.1
Trop	*LOC_Os04g56950*	4	33950221	33952563	3.4E+07	3.60E-06	8	4.65	8
Trop	*LOC_Os07g24190*	7	13741284	13747256	1.4E+07	2.60E-06	8.1	5.62	9.3
Trop	*LOC_Os12g12720*	12	7011245	7012771	7011126	–	–	3.25	5.6
Trop	*LOC_Os12g12720*	12	7011245	7012771	7011161	–	–	3.38	5.9
Trop	*LOC_Os12g12720*	12	7011245	7012771	7011295	–	–	3.69	6.7
Trop	*LOC_Os12g12720*	12	7011245	7012771	7012962	–	–	4.62	8
Indx	*LOC_Os02g17680*	2	10181426	10189201	1E+07	5.00E-05	5.4	3.63	6.5
Indx	*LOC_Os02g17680*	2	10181426	10189201	1.1E+07	8.10E-07	8.2	4.69	7.4
Indx	*LOC_Os04g56950*	4	33950221	33952563	3.4E+07	–	–	3.32	5.5
Indx	*LOC_Os06g24850*	6	14579528	14580059	1.5E+07	–	–	3.1	4.8
Indx	*LOC_Os07g40240*	7	24125333	24127487	2.4E+07	–	–	3.05	4.8
Indx	*LOC_Os07g40240*	7	24125333	24127487	2.4E+07			4.56	7.1

### Haplotype analysis for the significant genes

Haplotype analysis was performed for the six genes significantly associated with ML in Trop and Indx panel (**Table 3**). A total of 9 haplotypes of *LOC_Os02g17680* were identifiedin Trop and Indx panel, and named as *LOC_Os02g17680-Hap1-Hap9*. Of these, only *LOC_Os02g17680-Hap1*, *Hap2* and *Hap4* existed in Trop, whereas *LOC_Os02g17680-Hap1, Hap2, Hap3, Hap4, Hap5, Hap6, Hap7, Hap8* and *Hap9* l existed in Indx. A total of 9 haplotypes of *LOC_Os04g56950* were identified and named as *LOC_Os04g56950-Hap1-Hap9*. Of these, *LOC_Os04g56950-Hap1*, *Hap2*, *Hap3*, *Hap6* and *Hap8* distributed in Trop panel, whereas *LOC_Os04g56950-Hap1*, *Hap3*, *Hap4*, *Hap5*, *Hap7* and *Hap9* were existed in Indx panel. Totally, 3 haplotypes of *LOC_Os06g24850* were identified and named as *LOC_Os06g24850-Hap1-Hap3*. *LOC_Os06g24850-Hap1* and *Hap2* distributed in Trop panel, whereas *LOC_Os Os06g24850-Hap1*, *Hap2* and *Hap3* existed in Indx panel. *LOC_Os07g40240* including 3 haplotypes, e.g., *LOC_Os07g40240-Hap1~Hap3*. Of these, *LOC_Os07g40240-Hap1*, *Hap2* and *Hap3* distributed in Trop panel, whereas only *LOC_Os07g40240-Hap1* and *Hap3* were detected in Indx. Totally, *LOC_Os07g24190* including six haplotypes and named as *LOC_Os07g24190-Hap1-6.* Among these, only *Hap3* and *Hap6* distributed in Trop panel, whereas *Hap1-5* were identified in Indx panel. A total of 8 haplotypes of *LOC_Os12g12720* were identified in all accessions and named *LOC_Os12g12720-Hap1-Hap8*. Of these, *Hap1*, *Hap2*, *Hap3*, *Hap6* and *Hap7* of *LOC_ Os12g12720* distributed in Trop, whereas *LOC_Os12g12720*-*Hap1*, *Hap3*, *Hap4*, *Hap5* and *Hap8* were existed in Indx panel.

**Table 3:** The haplotype analysis and the superior haplotype for mesocotyl length in Trop and Indx panel.

Gene	Haplotype	Trop panel			Indx panel		
		Sample	Percentage	Mesocotyl length (cm)	Sample	Percentage	Mesocotyl length (cm)
			(%)			(%)	
*LOC_Os02g17680*	*Hap1*	250	75.5	1.89a	35	7.4	1.33b
	*Hap2*	5	1.2	1.97a	71	15.1	1.61a
	*Hap3*	–	–	–	71	15.1	1.27b
	*Hap4*	45	13.6	1.28b	67	14.3	1.28b
	*Hap5*	–	–	–	45	9.6	1.69a
	*Hap6*	–	–	–	20	4.3	1.42b
	*Hap7*	–	–	–	17	3.6	1.66a
	*Hap8*	–	–	–	10	2.1	1.21b
	*Hap9*	–	–	–	9	1.9	0.65c
*LOC_Os04g56950*	*Hap1*	98	29.6	2.00a	264	56.2	1.3b
	*Hap2*	82	24.8	1.99a	–	–	–
	*Hap3*	35	10.6	1.13c	12	2.6	1.58b
	*Hap4*	–	–	–	26	5.5	2.12a
	*Hap5*	–	–	–	19	4	1.08bc
	*Hap6*	12	3.6	1.61b	–	–	–
	*Hap7*	–	–	–	12	2.6	0.73c
	*Hap8*	9	2.7	2.02a	–	–	–
	*Hap9*	–	–	–	7	1.5	0.73c
*LOC_Os06g24850*	*Hap1*	60	18.1	1.78	438	93.2	1.35b
	*Hap2*	258	77.9	1.8	12	2.6	1.42a
	*Hap3*	–			5	1.1	1.34b
	*Hap1*	15	4.5	0.99	413	87.9	1.34b
*LOC_Os07g40240*	*Hap2*	294	88.2	1.82	–	–	–
	*Hap3*	5	1.2	1.96	11	2.3	1.41a
*LOC_Os07g24190*	*Hap1*	–	–	–	272	57.9	1.37
	*Hap2*	–	–	–	97	20.6	1.2
	*Hap3*	292	88.2	1.82a	20	4.3	1.07
	*Hap4*	–	–	–	13	2.8	1.29
	*Hap5*	–	–	–	7	1.5	2.18
	*Hap6*	12	3.6	1.13b	–	–	–
*LOC_Os12g12720*	*Hap1*	7	2.1	1.75b	230	48.9	1.24
	*Hap2*	181	54.7	1.78b	–	–	–
	*Hap3*	31	9.4	1.92a	61	13	1.7
	*Hap4*	–	–	–	50	10.6	1.02
	*Hap5*	–	–	–	41	8.7	2
	*Hap6*	37	11.2	1.86a	–	–	–
	*Hap7*	33	10	1.63c	–	–	–
	*Hap8*	–	–	–	23	4.9	1.18

The highest haplotype frequency in Trop were recorded in *LOC_Os02g17680-Hap1* (75.5%), *LOC_Os04g56950-Hap2* (29.6%), *LOC_Os07g24190-Hap3* (88.2%) and *LOC_Os12g12720-Hap2* (54.7%); whereas the lowest in Trop were recorded in *LOC_Os02g17680-Hap2* (1.2%), *LOC_Os04g56950-Hap8* (2.7%), *LOC_Os07g24190-Hap6* (3.6%) and *LOC_Os12g12720-Hap1* (2.1%). The highest haplotype frequency in Indx were recorded in *LOC_Os02g17680-Hap2* (15.1%) and *Hap3* (15.1%), *LOC_Os04g56950-Hap1* (56.2%), *LOC_Os06g24850-Hap1* (93.2%), *LOC_Os07g40240-Hap1* (87.9%); whereas the lowest in Indx were recorded in *LOC_Os02g17680-Hap9* (1.9%), *LOC_Os04g56950-Hap9* (1.5%), *LOC_Os06g24850-Hap3* (1.1%) and *LOC_Os07g40240-Hap3* (2.3%).

### The identification of superior haplotypes

To reduce the noise originated from population structure, the Duncan’s-test was established to identify the superior haplotypes of Trop and Indx separately (**Table 3**). According to the **Table 3** and **Figure S2**, 8 superior haplotypes were identified in *Trop* panel (**Figure S2**), including *LOC_Os02g17680-Hap1* (1.89* cm*) and *Hap2* (1.97* cm*), which significantly long than *Hap4* (1.28* cm*); *LOC_Os04g56950-Hap1* (2.00 cm), *Hap2* (1.99 cm) and *Hap8* (2.02* cm*) were significantly longer than *Hap3* (1.13 cm) and *Hap6* (1.61 cm); *LOC_Os07g24190-Hap3* (1.82* cm*) with longest mesocotyl relative to the *Hap6* (1.13 cm), *Hap1* (1.75 cm), *Hap2* (1.78 cm) and *Hap7* (1.63 cm) of *LOC_Os12g12720* were significantly shorter than *Hap3* (1.92 cm) and *Hap6* (1.86* cm*) (*P*<0.05). Six superior haplotypes were identified in Indx panel, including the *LOC_Os02g17680-Hap2* (1.61 cm)*, Hap5* (1.69 cm) and *Hap7* (1.66 cm) were significantly longer than *Hap1* (1.33 cm), *Hap3* (1.27 cm), *Hap4* (1.28 cm), *Hap6* (1.42 cm), *Hap8* (1.21 cm) and *Hap9* (0.65 cm). *LOC_Os04g56950-Hap4* (2.12* cm*), which were significantly longer than *Hpa1* (1.30 cm), *Hap3* (1.58* cm*), *Hap5* (1.08 cm), *Hap7* (0.73 cm) and *Hap9* (0.73 cm). For *LOC_Os06g24850-Hap2* (1.42* cm*), *Hap2* (1.42 cm) showed longer mesocotyl than *Hap1* (1.35 cm) and *Hap3* (1.34 cm). Furthermore, *LOC_Os07g40240-Hap3* (1.45* cm*) is higher than *Hap1* (1.34 cm) (**Figure S2**) (*P*<0.05).

According to the data from RFGB database, the seedling height of superior haplotypes *LOC_Os02g17680-Hap2* (36.4* cm*) is higher than that of *LOC_Os02g17680-Hap1* (35.6* cm*) and *LOC_Os02g17680-Hap4* (35.3* cm*)*; LOC_Os04g56950-Hap1* (36.4 cm), *Hap2* (35.2 cm) and *Hap8* (35.6* cm*) is higher than other haplotypes (32.0-35.5 cm) in Trop. However, the seedling height of superior haplotypes *LOC_Os07g24190-Hap3* (34.5* cm*), *LOC_Os12g12720-Hap3* (34.1 cm) and *Hap6* (33.9* cm*) is lower than other haplotypes. In *Trop*, the seedling height of superior haplotypes *LOC_Os02g17680-Hap2* (43.2 cm)*, Hap5* (41.9 cm) and *Hap7* (39.3 cm), *LOC_Os04g56950-Hap4* (37.69* cm*) and *LOC_Os07g40240-Hap3* (2.30%, 1.45 cm) is higher than other correspondence un-superior haplotypes; whereas *LOC_Os06g24850-Hap2* (37.3* cm*) is lower than correspondence un-superior haplotypes.

**Figure 3:**
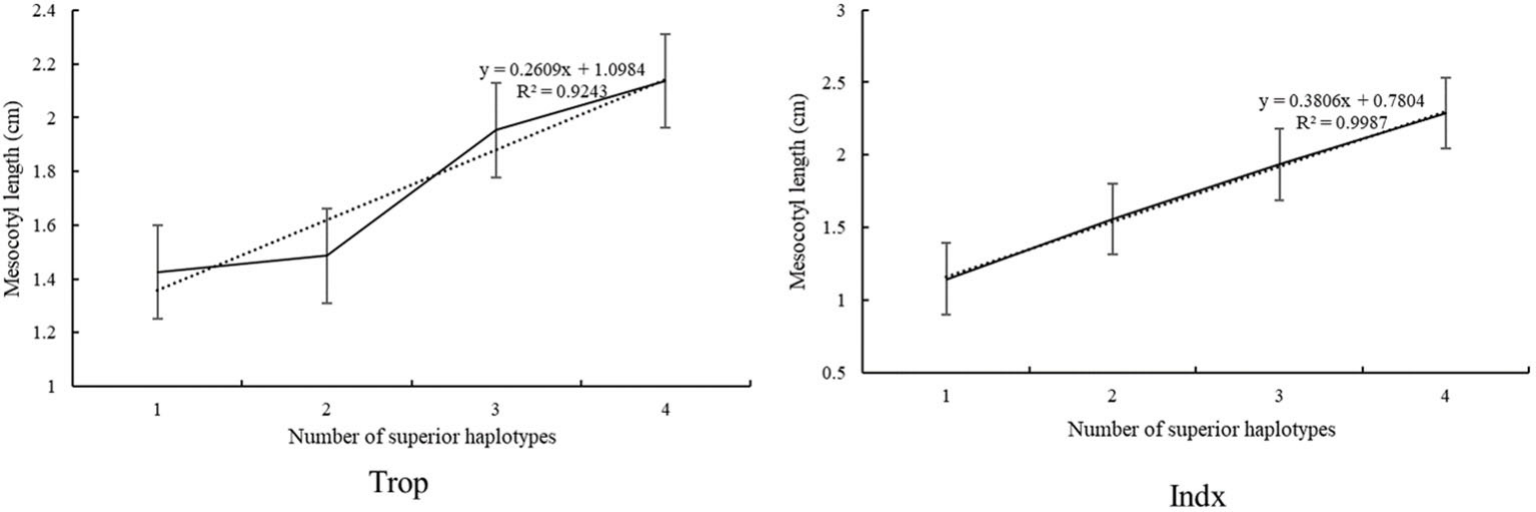
The linear regression between the number of superior haplotypes and mesocotyl length.

To further understand the additive effects of haplotypes on ML, we examined the number of superior haplotypes in each accession of Trop and Indx panel. The ML ranged from 1.41 cm to 2.15 cm with the superior haplotypes ranged from 0 to 3 in the Trop, whereas the ML ranged from 1.15 cm to 2.34 cm with the number of superior haplotypes ranged from 0 to 3 in the Indx. The relationships between ML and the numbers of superior haplotypes estimated by linear regression showed a dependence of ML on the number of superior haplotypes in both panels (**Figure 3**).

## Discussion

The existence of considerable genetic and phenotype variations for ML has been observed in this study (Wu et al., 2005; Wu et al., 2015; Liu et al., 2020). Thus, evaluate the genetic effects and identify superior haplotypes is urgent and important for ML improvement. Long mesocotyl breeding is feasible and has great potential. Although a series of genomic regions and candidate genes for ML have been reported, their availability in rice breeding remains unclear. Deeper insights into the complex relationship among ML and identify corresponding candidate genes would greatly aid in the selection of appropriate genes and superior haplotypes. In this study, CAS based on 281 selected genes were separately conducted to identify the genes and corresponding superior haplotypes for ML.

Conventional SL-MLM have been widely applied to identify genetic variants in crops (Liu et al., 2017; Liu et al., 2020). However, SL-MLM have disadvantages as they ignore the overall effects of multiple minor loci, and suffer from multiple test corrections for critical values (Wen et al., 2018; Wang et al., 2020; Zhang et al., 2020). Differing from SL-MLM, all the potentially associated markers are selected by a random-SNP-effect MLM with a modified Bonferroni correction for significance test by mr-MLM. In this study, more loci for ML were identified by mr-MLM in both Trop and Indx panels. For example, *LOC_Os12g12720* was only detected by the mr-MLM in Trop; *LOC_Os04g56950*, *LOC_Os06g24850* and *LOC_Os07g40240* were only detected by mr-MLM in Indx. These data illustrate that mr-MLM is more effective and powerful to detect minor gene/loci for quantitative inheritance complex traits (Segura et al., 2012; Cui et al., 2018; Wen et al., 2018; Zhang et al., 2020b). The reason for the higher effective of mr-MLM maybe the two-step association analysis of statistical model and the relatively loose threshold (Zhang et al., 2020b).

The distributions of haplotypes for the same gene were different in the Trop and Indx. Previous studies have reported that haplotype distributions differ across various populations (Qian et al., 2017; Wang et al., 2019; Liu et al., 2021). For example, in the present study, all the 9 haplotypes of *LOC_Os02g17680* were existed in the Indx, whereas *Hap1*, *Hap2* and *Hap4* were only identified in the Trop; for *LOC_Os04g56950*, *Hap1* and *Hap3* distributed in both two panels, *Hap2*, *Hap6* and *Hap8* were only detected in Trop, whereas *Hap4*, *Hap5*, *Hap7* and *Hap9* detected in Indx panel. The frequencies of haplotype distribution in Trop and Indx were different. The frequency of *LOC_Os02g17680-Hap1* accounted for 75.5% in the Trop, whereas 7.4% in Indx; the frequency of *LOC_Os04g56950-Hap1* was about 29.6% in the Trop panel, whereas about 56.2% in Indx.

Plant hormones, such as SLs, CTK, ABA, BR, IAA and JAS have direct influence on mesocotyl elongation by affecting cell division or elongation*. LOC_Os06g24850* on chromosome 6 belonged to *OsIAA22*-Auxin-responsive gene family. Auxin based on the indole ring plays crucial roles in plant growth and development, such as the cell differentiation, division and elongation (Xu and Xue, 2012). Feng et al. (2017) have reported that the exogenous IAA could promote mesocotyl elongation of rice seedings after germination under darkness. *LOC_Os07g40240* on chromosomes 7 encodes the GASR9-Gibberellin-regulated GASA/GAST/Snakin family protein precursor. Liang et al. (2016) reported that the destabilization of cortical microtubules (CMTs) increased the GA level and further promote the mesocotyl elongation, while polymerization of CMT showed opposite effect by influencing the expression of *GA20ox2*, *GA3ox2* and *GID1* in GA biosynthesis. *LOC_Os02g17680* on chromosomes 2 is an ethylene-responsive related protein. *LOC_Os04g56950* and *LOC_Os12g12720* on chromosome 4 and 12 encoding jasmonate O-methyltransferase and jasmonate-induced protein, respectively. ETH works as a signal to regulate cell elongation through JA biosynthesis pathway. Xiong et al. (2017) reported that the *GY1* functions at the initial step of JA biosynthesis to repress mesocotyl and coleoptile elongation in etiolated rice seedings. ETH inhibits the expression of *GY1* in the JA biosynthesis pathway and enhance mesocotyl and coleoptile growth by promoting cell elongation (Xiong et al., 2017). *LOC_Os07g24190* on chromosome 7 encoding the CESA3-cellulose synthase, plays crucial roles in the roots, stems, and the elongation of root hair (Li et al., 2019; Moon et al., 2019).

Several studies have shown that mesocotyl has a significant impact on seedling height, and long mesocotyl accessions tend to with higher seedling height (Kumar and Ladha, 2011; Lee et al., 2017). This study verified the above results. Most of the superior haplotypes with higher seedling height, such as *LOC_Os02g17680-Hap2*, *LOC_Os04g56950-Hap1*, *Hap2* and *Hap8* in Trop panel, *LOC_Os02g17680-Hap2, Hap5* and *Hap7*, *LOC_Os04g56950-Hap4* and *LOC_Os07g40240-Hap3* in Indx panel. However, we also identified few ML superior haplotypes with shorter seedling height, such as the *LOC_Os07g24190-Hap3*, *LOC_Os12g12720-Hap3* and *Hap6* in Trop panel and *LOC_Os06g24850-Hap2* in the Indx. In rice breeding, seedling height selection is time-consuming and laborious, while mesocotyl phenotype evaluation can be carried out rapidly with high throughput. From the above results, in future rice breeding, superior haplotype accessions can be selected based on mesocotyl length, and then accessions with higher seedling height can be selected indirectly although seedling height is influenced by various factors besides mesocotyl length. However, these ML superior haplotypes with lower seedling height need to be specifically selected according to the breeding goal.

We examined the number of superior haplotypes in each accession to further understand the combined effects of alleles on ML. The ML ranged from 1.41 to 2.15 cm with the superior haplotypes ranged from 0 to 3 in the Trop panel, whereas the ML ranged from 1.15 to 2.34 cm with the superior haplotypes ranged from 0 to 3 in the Indx. A significant additive effect was identified from the linear regression between ML and the number of superior haplotypes, indicating that pyramiding of superior haplotypes will accelerate the genetic improvement of ML. As the distribution of superior haplotypes are different, genes and corresponding haplotypes should be selected specific for Trop and Indx. *LOC_Os02g17680* (*Hap1* (1.89 cm) and *Hap2* (1.97 cm) of Trop; *Hap2* (1.61 cm), *Hap5* (1.69 cm), and *Hap7* (1.66 cm) of Indx) and *LOC_Os04g56950* (*Hap1* (2.00 cm), *Hap2* (1.99* cm*) and *Hap8* (2.02) cm of Trop; *Hap4* (2.12 cm) of Indx) were detected in both Trop and Indx, implying that these genes play a stabilizing role in diverse accessions and could be widely used in rice breeding. *LOC_Os07g24190* (Hap3 1.82 cm) and *LOC_Os02g17680* (*Hap2* (1.61 cm), *Hap5* (1.69 cm), and *Hap7* (1.66 cm)) explained the highest phenotypic variations and is the best choice for higher ML breeding in Trop and Indx panels, respectively. Furthermore, *LOC_Os07g24190* (Hap3 1.82 cm) and *LOC_Os12g12720* (Hap3 1.70 cm) could be applied in the Trop panel specifically, whereas the *LOC_Os06g24850* (1.42 cm) and *LOC_Os07g40240* (1.41 cm) could be used in Indx panel specifically. *LOC_Os02g17680-Hap1* (1.89 cm) and *Hap2* (1.97* cm*), *LOC_Os04g56950-Hap1* (2.00* cm*), *Hap2* (1.99 cm) and *Hap8* (2.02* cm*), *LOC_Os07g24190-Hap3* (1.82* cm*), *LOC_Os12g12720-Hap3* (1.92 cm) and *Hap6* (1.86 cm) are recommended for ML improvement in Trop, whereas the *LOC_Os02g17680-Hap2* (1.61* cm*), *Hap5* (1.69 cm) and *Hap7* (1.66* cm*), *LOC_Os04g56950-Hap4* (2.12* cm*), *LOC_Os06g24850-Hap2* (1.42 cm) and *LOC_Os07g40240-Hap3* (1.41 cm) are suitable in Indx. Lines with higher ML and carrying multiple superior haplotypes, such as SUNGKAI, RIMBUN, SINAPLED, YAH YAW, IRAT 104/PALAWAN, BIKYAT and BUNTU DOMBA 1 in Trop, ARC 14064, CHNNOR, ARC 11857, BAIANG 6, LANJALI and JIA GEN in Indx could be used to rapidly combine several superior target haplotypes into one background.

## Conclusion

In the present study, 281 ML related genes were selected to evaluate their effects for ML and identify superior haplotypes in two different populations. Totally, six unique genes were identified for ML. Of these, *LOC_Os02g17680* and *LOC_Os04g56950* were identified in both two panels. Totally, 8 and 6 superior haplotypes for ML were identified in Trop and Indx panel, respectively. A significant additive effect was identified from the linear regression between ML and the number of superior haplotypes. Introgression of these superior haplotypes by the haplotype‐based breeding is a promising strategy. The associated genes and superior haplotypes may pave the way for future rice ML breeding.

## Data availability statement

The original contributions presented in the study are included in the article/**Supplementary Material**. Further inquiries can be directed to the corresponding authors.

## Author contributions

JL and GY designed the research, analyzed the physiology data, YW and JL drafted the manuscript. YM and HL performed the experiment. JL revised the manuscript. All authors have read, edited and approved the current version of the manuscript.

## Supplementary material

Supplementary Figure 1 The distribution of mesocotyl length and seedling height in Trop and Indx panels.

Supplementary Figure 2 Comparison of mesocotyl length between superior haplotypes and other haplotypes for the significant genes in the *Trop* and *Indx* panel. Different letters represent significant differences at the P=0.05 level.
