# Supplementary figures and images for: Validation of genes affecting rice mesocotyl length through candidate association analysis and identification of the superior haplotypes
Source: Front Plant Sci. 2023 May 30;14:1194119. doi: 10.3389/fpls.2023.1194119 (PMC10267709; doi:10.3389/fpls.2023.1194119)

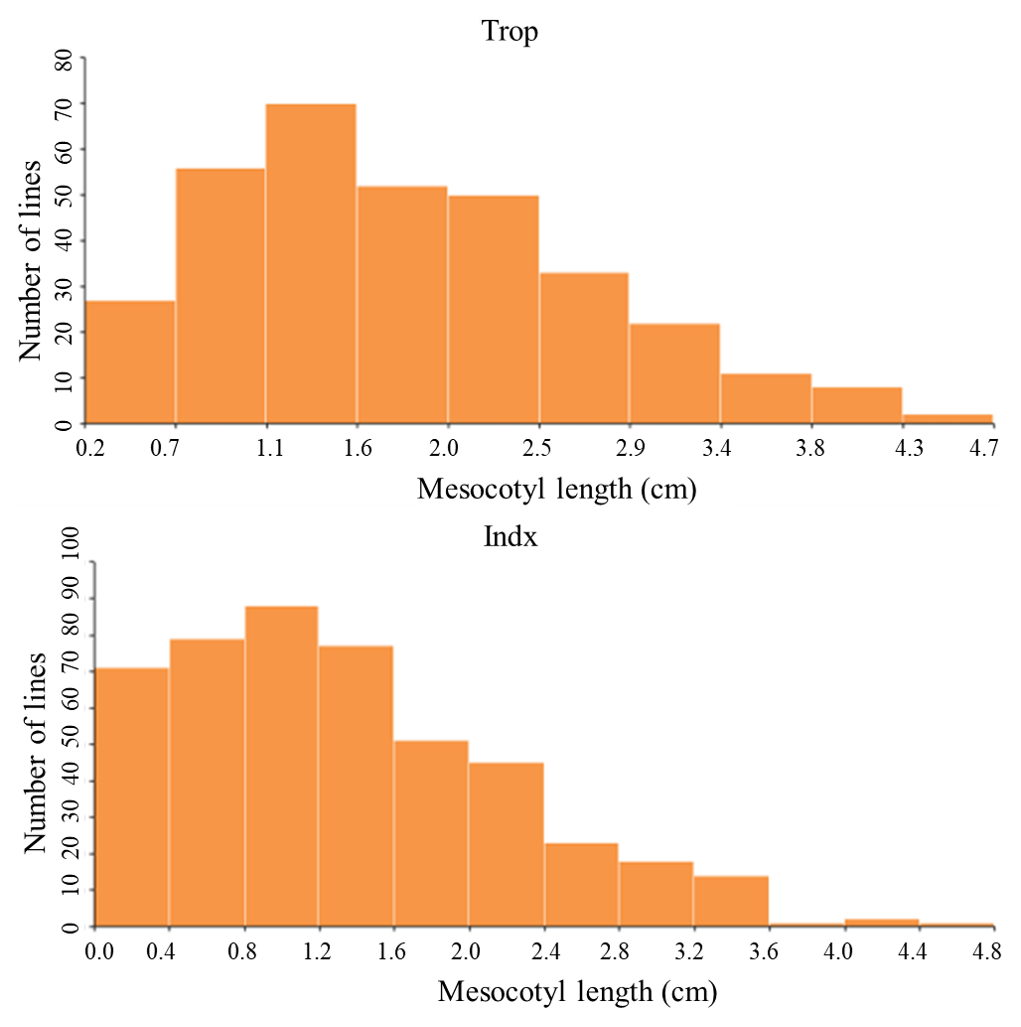

Supplement: Supplementary Figure 1 — The distribution of mesocotyl length and seedling height in Trop and Indx panels. [file Image_1.tif]

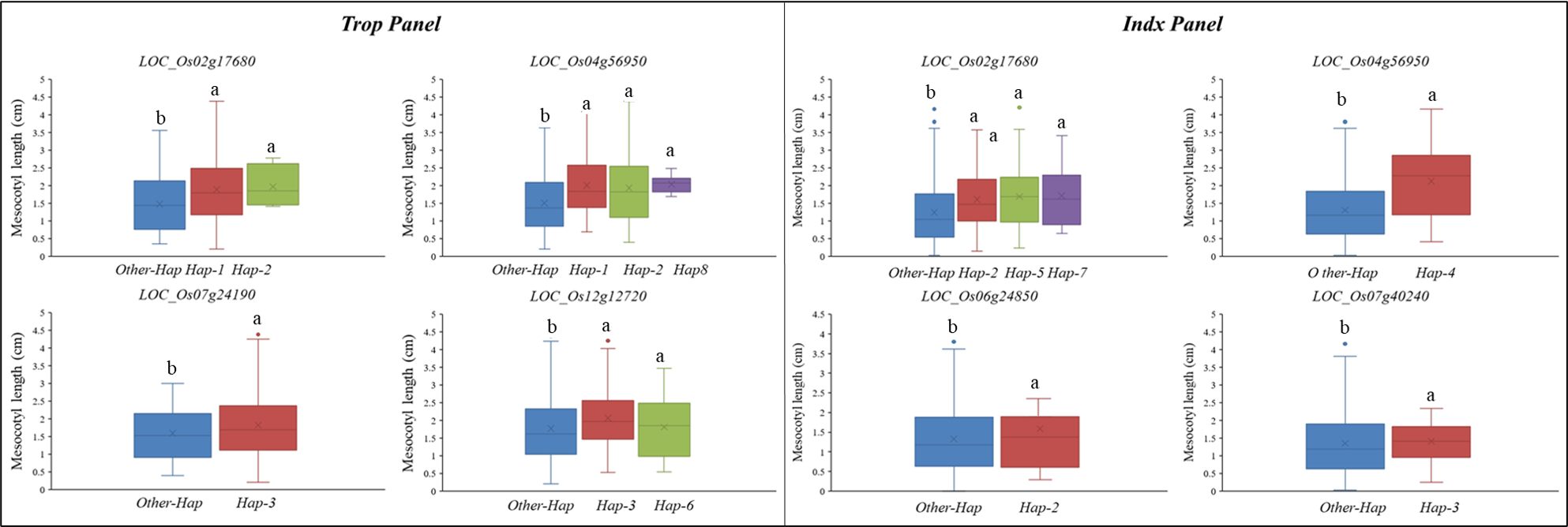

Supplement: Supplementary Figure 2 — Comparison of mesocotyl length between superior haplotypes and other haplotypes for the significant genes in the Trop and Indx panel. Different letters represent significant differences at the P=0.05 level. [file Image_2.tif]
